# Long non-coding RNA MIAT promotes growth and metastasis of colorectal cancer cells through regulation of miR-132/Derlin-1 pathway

**DOI:** 10.1186/s12935-017-0477-8

**Published:** 2018-04-16

**Authors:** Zhaoxia Liu, Hai Wang, Hongwei Cai, Ye Hong, Yan Li, Dongming Su, Zhining Fan

**Affiliations:** 1 Medical Center for Digestive Diseases, the Second Affiliated Hospital of Nanjing Medical University, Nanjing, China; 2 Department of Gastroenterology, Affiliated Nanhua Hospital, University of South China, Hengyang, China; 3 Center of Pathology and Clinical Laboratory, Sir Run Run Hospital, Nanjing Medical University, Nanjing, China; 4 Department of Pathology, Nanjing Medical University, Nanjing, China; 5 Digestive Endoscopic Center, the First Affiliated Hospital of Nanjing Medical University, Nanjing, China

**Keywords:** Colorectal cancer, MIAT, miR-132, Derlin-1

## Abstract

**Objective:**

Recently, long non-coding RNA (lncRNA) MIAT has been demonstrated as an oncogenic gene in several types of cancer. However, the role and mechanism of MIAT in colorectal cancer (CRC) have not been investigated.

**Methods:**

Real-time PCR was used to measure MIAT expression in CRC tissues and cells. Small interfering RNA specific for MIAT (si-MIAT) was used to down-regulate MIAT expression in CRC cells. The interaction of MIAT and miR-132 was measured by RNA pull-down assay. The effect of si-MIAT on CRC cells apoptosis and metastasis were measured by flow cytometry assay, invasion and migration assay, respectively.

**Results:**

In present study, we found that MIAT was highly expressed in CRC tissues and cells. MIAT knockdown inhibited proliferation, migration and invasion and enhanced apoptosis of CRC cells. Further, we demonstrated that MIAT acted as a competing endogenous RNA for miR-132, antagonized its functions, and resulted in the de-repression of its target gene Derlin-1, which acted as an oncogene in promoting growth and metastasis of CRC cells. In LOVO and SW480 cells with si-MIAT, miR-132 inhibitor resulted in an increase of cell proliferation, migration and invasion and a decrease of cell apoptosis, which was partially abolished by transfection of Derlin-1 shRNA.

**Conclusions:**

Our data indicated that highly expressed MIAT was an oncogenic lncRNA that promoted the growth and metastasis of CRC through miR-132/Derlin-1 axis.

**Electronic supplementary material:**

The online version of this article (10.1186/s12935-017-0477-8) contains supplementary material, which is available to authorized users.

## Background

Colorectal cancer (CRC) is one of the most common gastrointestinal malignancy worldwide, with over one million newly diagnosed cases every year [1]. The 5-year survival rate in CRC patients with early-stage is over 90% but decrease to less than 10% in patients with advanced disease and metastasis [2]. Thus, it is of great importance to make clear the key molecules closely associated with proliferation and metastasis of CRC cells. Recently, more and more evidences have indicated that non-coding RNAs including miRNAs and lncRNAs act as modulators in CRC cell growth and metastasis [3, 4].

MicroRNA-132 (miR-132) has been investigated to be involved in the development and progression of a series of cancers. In CRC, miR-132 functions as a tumor suppressor. The decreased miR-132 level was observed in CRC tissues and was associated with tumor size, TNM stage, distant metastasis and poor survival in CRC patients [5, 6]. Further study showed that over-expression of miR-132 could inhibit EMT and invasion, while down-regulation of miR-132 increased CRC cell motility and EMT progression [7]. Therefore, miR-132 might serve as a prognostic indicator and therapeutic candidate in CRC patients.

Long non-coding RNA myocardial infarction associated transcript (lncRNA-MIAT) is located in the nucleus and acts on regulating gene expression [8]. MIAT has been confirmed to be associated with myocardial infarction, diabetes-related diseases, paranoid schizophrenia and cancer [9]. In recent years, the mechanism of the interaction between lncRNAs and microRNAs has been drawn attention during the tumorigenic process. Li et al. discovered that high expression of lncRNA-MIAT in Lung adenocarcinoma increases the expression of miR-106 target gene MAPK9 by competitively ‘spongeing’ miR-106, ultimately activates MAPK signaling pathways [10]. However, the role and mechanism of lncRNA-MIAT in CRC cell growth and metastasis remains unclear.

In this study, we first measured lncRNA-MIAT expression in CRC. The results showed that the level of lncRNA-MIAT was significantly elevated in CRC tissues and cells. Furthermore, we showed that knock-down of MIAT induced apoptosis and inhibited metastasis of CRC cells. The down-regulation of lncRNA-MIAT resulted in the increase of miR-132 expression and the decrease of Derlin-1 expression. Taken together, these results showed that lncRNA-MIAT played an important role in regulating the growth and metastasis of colon cancer.

## Results

### LncRNA-MIAT expression was up-regulated in CRC tissues and cell lines

**Fig. 1:**
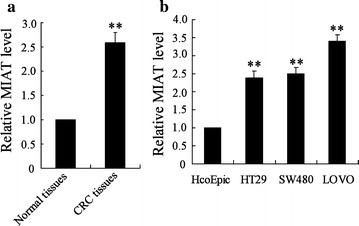
LncRNA-MIAT expression was up-regulated in CRC tissues and cell lines. **a** LncRNA-MIAT expression was measured by real-time PCR in 30 pairs of CRC tissue and matched adjacent normal tissue samples. **b** LncRNA-MIAT expression was measured by real-time PCR in HcoEpic, HT-29, SW480 and LOVO cells. **P < 0.01, compared to normal tissues or HcoEpic cells

Real-time PCR analysis was used to determine lncRNA-MIAT expression in 30 pairs of CRC tissue and matched adjacent normal tissue samples. As shown in Fig. 1a, MIAT was up-regulated in CRC tissue compared with adjacent normal tissues. In addition, we also measured MIAT expression in HcoEpic HT-29, SW480 and LOVO cells. Our results showed that MIAT expression was significantly increased in CRC cells (Fig. 1b).

### Down-regulation of MIAT inhibited CRC cell proliferation, migration and invasion

**Fig. 2:**
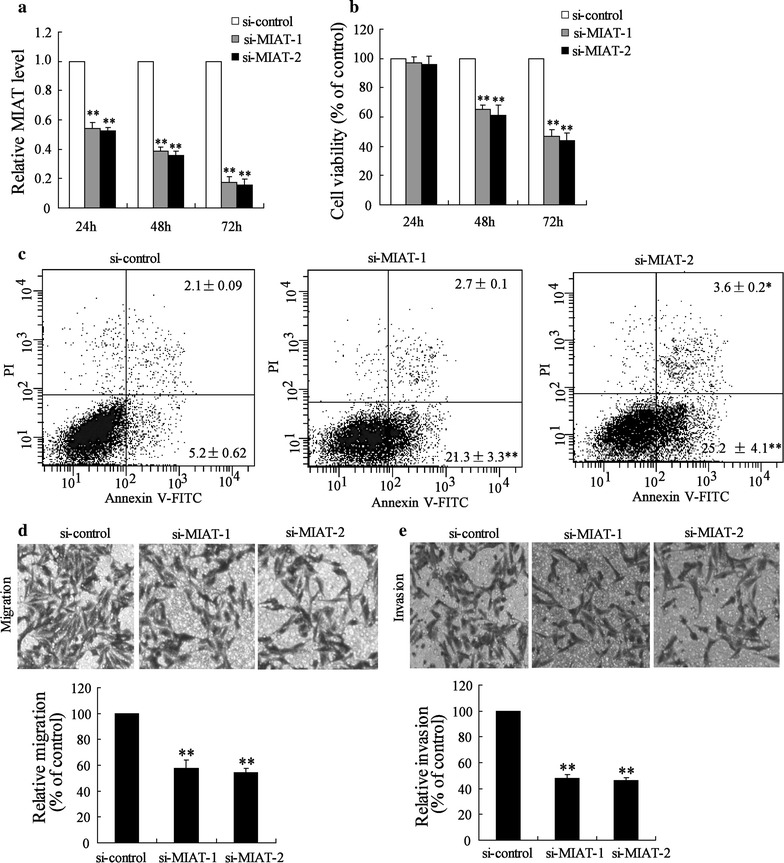
Down-regulation of MIAT inhibited CRC cell proliferation, migration and invasion. LOVO cells were transfected with si-control, si-MIAT-1 or si-MIAT-2 for different time, **a** MIAT expression and (**b**) cell viability was measured. LOVO cells were transfected with si-control, si-MIAT-1 or si-MIAT-2 for 72 h, **c** cell apoptosis, **d** cell migration and **e** cell invasion was determined. **P < 0.01, compared to si-control

To investigate the role of MIAT in colon cancer cells, endogenous MIAT expression was inhibited by si-MIAT in LOVO cells, which have high endogenous MIAT levels. The result showed that si-MIAT-1 and si-MIAT-2 could significantly inhibit MIAT expression (Fig. 2a). We then explored the effect of si-MIAT on the growth of LOVO cells. Cell proliferation assay revealed that si-MIAT-1 and si-MIAT-2 inhibited growth of LOVO cells in a time-dependent manner (Fig. 2b). The SW480 cells with decreased expression of MIAT showed significantly inhibited proliferation (Additional file 1: Figure S1A). Furthermore, we determined the effects of MIAT on the apoptosis of colon cells by flow cytometry. As shown in Fig. 2c, the rate of apoptotic cells in si-control, si-MIAT-1 and si-MIAT-2 transfected LOVO cells were 5.2, 21.3 and 25.2%, respectively. Knockdown of MIAT significantly increased the apoptosis of SW480 cells (Additional file 1: Figure S1B). However, si-MIAT-1 and si-MIAT-2 had no effect on cell cycle (data not shown). These results suggested that the decrease in the number of cells upon si-MIAT transfection was caused by cell apoptosis.

Next, we performed migration and invasion assays in LOVO cells transfected with si-MIAT-1 and si-MIAT-2. Results demonstrated knockdown of MIAT reduced cell migration to 58 and 54% using si-MIAT-1 and si-MIAT-2, respectively (Fig. 2d). Similarly, LOVO cell invasion was also reduced to 48 and 46% after treating with si-MIAT-1 and si-MIAT-2, respectively (Fig. 2e). Knockdown of MIAT significantly reduced migration and invasion of SW480 cells (Additional file 1: Figure S1C). Taken together, these results indicated that MIAT may play oncologic role through promoting cell proliferation, migration and invasion, and inhibiting cell apoptosis in CRC.

### MIAT functioned as miR-132 sponge in colon cancer cells

**Fig. 3:**
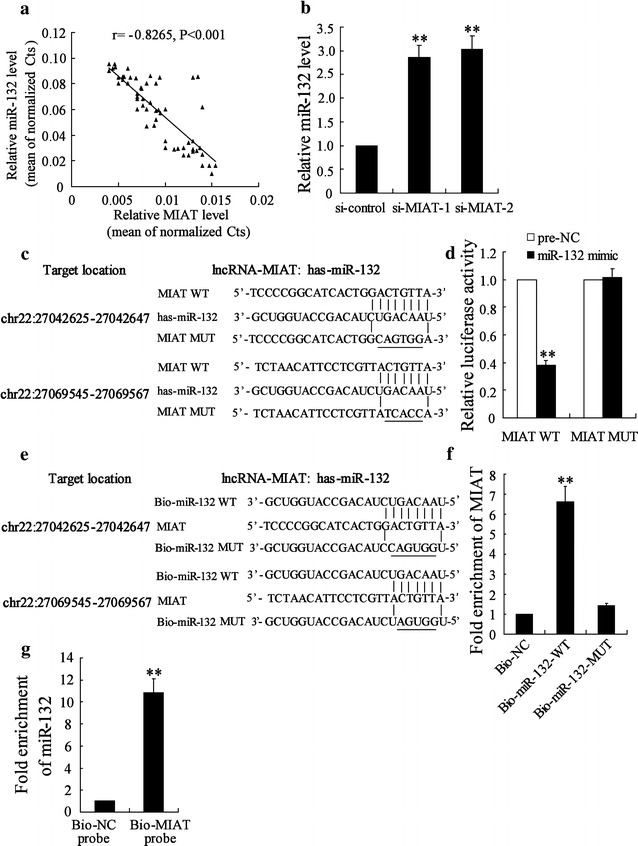
MIAT functioned as miR-132 sponge in colon cancer cells. **a** The correlation between MIAT-1 and miR-132 expression in 30 CRC tissues. **b** LOVO cells were transfected with si-control, si-MIAT-1 or si-MIAT-2 for 48 h, miR-132 expression was measured. **c** Sequence alignment of miR-132 with the putative binding sites within the wild-type regions of MIAT-1. **d** LOVO cells were co-transfected with miR-132 mimic and MIAT-1-WT vector or MIAT-1-MUT vector for 48 h, the luciferase activity was measured. **e** WT and the mutated forms of miR-132 sequence were shown. **f** Detection of MIAT using real-time PCR in the sample pulled down by biotinylated miR-132. **g** Detection of miR-132 using real-time PCR in the sample pulled down by biotinylated MIAT probe. **P < 0.01, compared to si-control, Pre-NC or Bio-NC

LncRNAs can act as competing endogenous RNAs or endogenous sponge RNAs to absorb miRNAs by sequence complementarity and in turn affecting miRNAs’ biological functions [11]. In the present study, we evaluated the association between MIAT and miR-132 expression in 30 CRC tissues, and results suggested the expression of MIAT and miR-132 showed a significantly negative correlation as analyzed by Pearson correlation analysis (r = − 0.8265, *P* < 0.0001) (Fig. 3a). Besides, knockdown of MIAT remarkably elevated miR-132 expression in LOVO cells (Fig. 3b).

A database (starBase2.0) revealed the significant sequence complementarity between MIAT and miR-132 (Fig. 3c). Luciferase reporter assay showed that overexpression of miR-132 could decrease MIAT-WT activity, while it had no effect on MIAT-MUT (Fig. 3d). Additionally, we performed a biotin-avidin pull-down assay to find out whether miR-132 could pull down MIAT. Our results suggested that MIAT was pulled down by miR-132, but the introduction of mutations which disrupted the predicted miRNA recognition sites between MIAT and miR-132 led to the inability of miR-132 to pull down MIAT (Fig. 3e, f), which indicated that the recognition of miR-132 to MIAT was in a sequence-specific manner. We also applied inverse pull-down assay using biotinylated lncRNA-MIAT DNA probe to investigate whether MIAT could pull down miR-132, and miR-132 was precipitated and analyzed by real-time PCR analysis (Fig. 3g). These results revealed that MIAT acted as miR-132 sponge in colon cancer cells.

### MiR-132 directly targeted oncogene Derlin-1

**Fig. 4:**
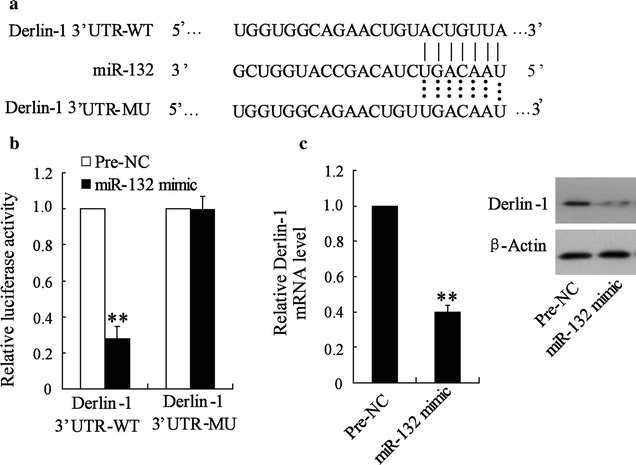
MiR-132 directly targeted oncogene Derlin-1. **a** The 3′-UTR of Derlin-1 harbored miR-132 cognate site. **b** LOVO cells were co-transfected with miR-132 mimic and wild-type or mutant Derlin-1 3′-UTR for 48 h, the luciferase activity was measured. **c** LOVO cells were transfected with Pre-NC or miR-132 mimic for 48 h, the mRNA and protein level of Derlin-1 was measured. **P < 0.01, compared to Pre-NC

Zheng et al. reported that miR-132 was significantly decreased in CRC tissues and cells, which acted as a tumor suppressor. Our previous study showed that Derlin-1 overexpressed in colon cancer and promoted proliferation of colon cancer cells [12]. TargetScan and miRanda revealed that the 3′-UTR of Derlin-1 contained the complementary site for the seed region of miR-132 (Fig. 4a). Further study showed that miR-132 overexpression could significantly reduce Derlin-1 3′-UTR activity, a finding that was not observed for the mutant Derlin-1 3′-UTR activity (Fig. 4b). Additionally, miR-132 overexpression could significantly decrease the Derlin-1 mRNA and protein level in LOVO cells (Fig. 4c). These results indicated that miR-132 targeted human Derlin-1 by directly binding to the predicted sites in 3′-UTR of Derlin-1 mRNA.

### MIAT regulated Derlin-1 expression through modulating miR-132

**Fig. 5:**
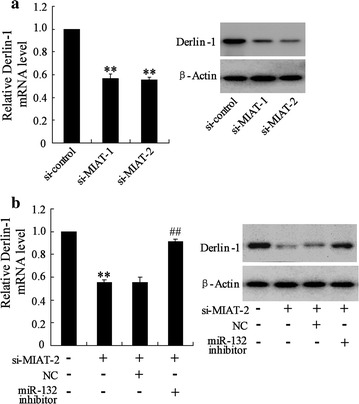
MIAT regulated Derlin-1 expression through modulating miR-132. **a** LOVO cells were transfected with si-control, si-MIAT-1 or si-MIAT-2 for 48 h, the mRNA and protein of Derlin-1 expression was measured. **b** LOVO cells were transfected with si-MIAT-2 and miR-132 inhibitor, the mRNA and protein of Derlin-1 expression was measured. **P < 0.01, compared to si-control. ^##^P < 0.01, compared to si-MIAT-2 + NC

Because MIAT shared regulatory miR-132 with Derlin-1 mRNA, we wondered whether MIAT could regulate Derlin-1 in CRC cells. As the result shown, down-regulation of MIAT expression decreased Derlin-1 mRNA and protein levels in LOVO cells (Fig. 5a). Furthermore, down-regulation of miR-132 upon si-MIAT transfection abrogated this decrease (Fig. 5b). All these results suggested an important role of MIAT in modulating Derlin-1 by competitively binding miR-132.

### Down-regulation of MIAT inhibited CRC cell proliferation, migration and invasion by miR-132/Derlin-1 axis

**Fig. 6:**
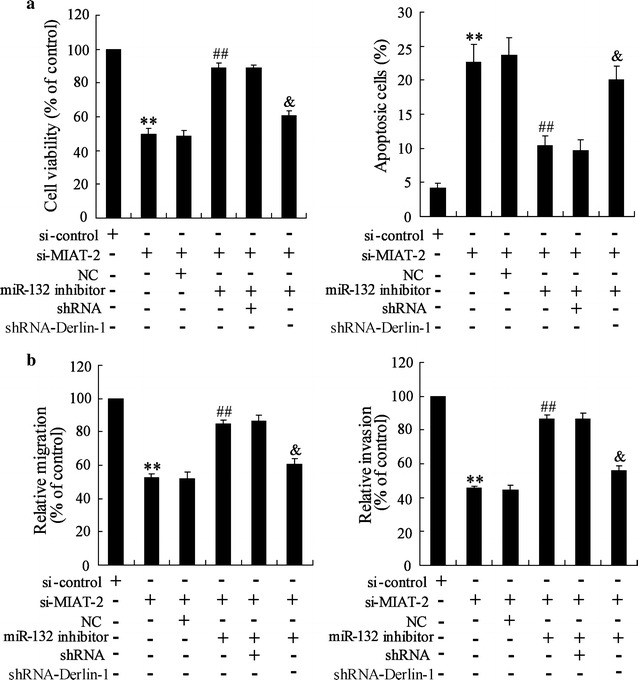
Down-regulation of MIAT inhibited CRC cell proliferation, migration and invasion by miR-132/Derlin-1 axis. LOVO cells were transfected with si-MIAT-2, miR-132 inhibitor and Derlin-1 shRNA (shRNA-Derlin-1) for 72 h, **a** cell viability, cell apoptosis, **b** cell migration and cell invasion was determined. **P < 0.01, compared to si-control. ^##^P < 0.01, compared to si-MIAT-2 + NC. ^&^P < 0.01, compared to si-MIAT-2 + miR-132 inhibitor + shRNA

To further explore whether down-regulation of MIAT inhibiting CRC cell proliferation, migration and invasion was mediated by miR-132, we performed rescue experiments. In LOVO cells with si-MIAT, miR-132 inhibitor resulted in a significant increase of cell proliferation and decrease of cell apoptosis, which was partially abolished by transfection of Derlin-1 shRNA (Fig. 6a). In addition, down-regulation of miR-132 induced an increase of migration and invasion in LOVO cells transfected with si-MIAT, which was also reversed by Derlin-1 shRNA (Fig. 6b). Similar results were also observed in SW480 cells (Additional file 2: Figure S2). These data suggested that down-regulation of MIAT inhibited CRC cell proliferation, migration and invasion which is mediated by miR-132**/**Derlin-1 axis.

## Discussion

Recent studies have revealed lncRNAs as functional regulators of several biological processes including growth and metastases in a variety of human tumors [13, 14]. Nevertheless, the role and mechanism of lncRNA-MIAT in CRC has not been thoroughly investigated. In present study, we provided new evidence that up-regulation of lncRNA-MIAT in CRC tissues and cell lines acted as oncogene. MIAT was revealed as miR-132 sponge in colon cancer cells. MIAT promoted CRC cell proliferation, migration and invasion in large part attributed to inhibit miR-132 (acting as competing endogenous RNAs) and subsequent regulation of Derlin-1 expression.

Recent studies found that lncRNA-MIAT played an oncogenic role in Lung adenocarcinoma, neuroendocrine prostate cancer and chronic lymphocytic leukemias [10, 15, 16]. In this study, we found MIAT expression in CRC tissues was significantly higher than that in normal colon tissues. MIAT expression was also increased in CRC cells compared to human normal colon cell line. More important, siRNA mediated MIAT knockdown inhibited proliferation, enhanced apoptosis and suppressed migration and invasion of LOVO cells. Collectively, our data suggested that MIAT may act as a carcinogenic role and could play a critical efficiency in CRC growth and metastasis.

It has been demonstrated that lncRNAs not only modulate proteins but also regulate the target gene of miRNA indirectly through affecting miRNA expression. Li et al. demonstrated that lncRNA-MIAT can act as competing endogenous RNAs (ceRNAs) to absorb miR-106 by sequence complementarity and in turn affecting miR-106′ biological functions in Lung adenocarcinoma [10]. In our present study, we also discovered that MIAT functioned as ceRNAs to bind to miR-132 and regulated its function. In CRC, miR-132 inhibited cell growth, metastasis, and induced cell apoptosis in colon cells [5–7]. Moreover, the expression of MIAT and miR-132 showed a significantly negative correlation in CRC tissues. And MIAT knockdown remarkably increased miR-132 expression. Biotin-avidin pull-down analysis demonstrated that MIAT could pull down miR-132. In addition, our data also found that miR-132 inhibitor could reverse the anti-cancer effects of MIAT knockdown on cell growth, metastasis and negative role of si-MIAT on cell apoptosis in CRC cells. Therefore, lncRNA-MIAT played its favorable role on CRC growth and metastasis, at least in part, through suppression of miR-132.

Derlin-1, a partner of the p97 ATPase complex, played an important role in cancer progression. Our previous study demonstrated that Derlin-1 was highly expressed in CRC and significantly correlated with tumor progression [12]. TargetScan and miRanda predicted that Derlin-1 may be a target of miR-132 and Starbase database indicated the target sites between MIAT and miR-132. Thus, we speculated that MIAT could regulate Derlin-1 thorough miR-132 network in CRC. Through luciferase reporter assay, we discovered that Derlin-1 is a direct target gene of miR-132. Moreover, we showed that MIAT silencing increased miR-132 level and decreased Derlin-1 expression level. These data were consistent with our predication and suggested that MIAT can regulate Derlin-1 through miR-132. Importantly, the effect of MIAT on CRC cell proliferation, migration and invasion is mediated by miR-132**/**Derlin-1 axis.

## Conclusions

Our data indicated that highly expressed MIAT was an oncogenic lncRNA that promoted the growth and metastasis of CRC through miR-132**/**Derlin-1 axis. The present data clarified a potential mechanism underlying the tumor-oncogenic role of lncRNA-MIAT in CRC, and indicated that lncRNA-MIAT could be a potential therapeutic target in CRC.

## Materials and methods

### Patients and tissue samples

Paired colon cancer and normal colon tissue were obtained between 2015 and 2016 from 30 patients who underwent primary surgical resection of colon cancer in Department of Gastroenterology of Affiliated Nanhua Hospital, University of South China (Hengyang, China). Follow-up information was obtained by reviewing patients’ medical records. None of the patients received radiotherapy or chemotherapy before surgical resection. All these tissue samples were immediately frozen in liquid nitrogen and stored at − 80 °C until total RNA and protein were extracted.

### Cell lines and cell culture

Human colon cancer cell lines HT-29, SW480, and LOVO and human normal colon cell line HcoEpic were purchased from the institute of Biochemistry and Cell Biology of the Chinese Academy of Sciences (Shanghai, China). Cells were cultured in Dulbecco’s Modified Eagle’s Medium (DMEM) or Roswell Park Memorial Institute 1640 (Hyclone, Logan, UT, USA) supplemented with 10% fetal bovine serum (FBS), 100 U/mL penicillin and 100 ng/mL streptomycin (Invitrogen, California, USA) in humidified air at 37 °C with 5% CO_2_.

### Real-time PCR assay

Total RNA of frozen tissues and cells were extracted using TRIzol reagent (Invitrogen, Grand Island, NY, USA) according to the manufacturer’s protocol. The expression of miR-132 and lncRNA-MIAT was quantified by real-time PCR using a LightCycler480 II Sequence Detection System (Roche, Basel, Switzerland). The specific primers were as follows: MIAT, 5′-TTTACTTTAACAGACCAGAA-3′ (forward) and 5′-CTCCTTTGTTGAATCCAT-3′ (reverse); GAPDH, 5′-GGGA GCCAAAAGGGTCAT-3′ (forward) and 5′-GAGTCCTTCCACGATACCAA-3′ (reverse); miR-132, 5′-GCCCGTAACAGTCTACAGCCAT-3′ (forward) and 5′-GCA GGGTCCGAGGTATTC-3′ (reverse); U6, 5′-GTGCGTGTCGTGGAGTCG-3′ (forward) and 5′-AACGCTTCACGAATTTGCGT-3′ (reverse). U6 and GAPDH were used as internal standard to normalize the miR-132 and lncRNA-MIAT expression level, respectively, using 2^−∆∆Ct^ method.

### Western blotting

Total protein from tissue and cells were extracted in lysis buffer (Vazyme Biotech, Nanjing, China) containing a protease inhibitor cocktail (Roche, Basel, Switzerland). Protein concentrations were determined using the Bio-Rad Protein Assay (Bio-rad, California, USA). Western blot analysis was performed as described [17]. Individual immunoblots were probed with a rabbit anti-Derlin-1 antibody (diluted 1:1000) and a mouse anti-β-Actin (diluted 1:3000).

### Small interfering RNA

Small interfering RNA specific for MIAT (si-MIAT) and control siRNA (si-NC) was synthesized (Ribobio, Guangzhou, China) and transfected using Lipofectamine 2000 in HT-29, SW480 and LOVO cells. The sequences of siRNA were: si-MIAT1, 5′-CCAGGCUCCUUUAAACCAATT-3′; si-MIAT2, 5′-GCAGUUCUUAGCUCAUA UATT′.

### Cell proliferation assay

The si-MIAT or si-NC-transfected cells were allowed to grow in 48-well plates and cultured for 48 h. At each time point, cells were stained with Cell Proliferation Reagent Kit I (MTT; Roche Applied Science, Basel, Switzerland) for 4 h at 37 °C. DMSO was used to dissolve the formazan, and the absorbance at 490 nm was measured using a microplate reader.

### Cell apoptosis assay

The si-MIAT or si-NC-transfected LOVO and SW480 cells were cultured in six-well plates for 48 h. The cells were harvested by trypsinization. Following double staining with FITC-annexin V and propidium iodide (PI), the cells were analyzed using flow cytometry (FACScan; BD Biosciences, San Jose, CA) as described previously [18].

### Invasion and migration assay

For the migration assays, 1 × 10^5^ colon cancer cells in serum-free media were placed into the upper chamber of a Transwell insert (8-μm pore size; Millipore). For the invasion assays, colon cancer cells in serum-free medium were placed into the upper chamber of an insert coated with Matrigel (Sigma-Aldrich). Medium containing 10% FBS was added to the lower chamber. After incubation for 16 h, the cells remaining on the upper membrane were removed with cotton wool. Colon cancer cells that had migrated or invaded through the membrane were fixed in methanol, stained with crystal violet (0.04% in water; 100 μL), counted using an inverted microscope and photographed.

### Pull-down assay with biotinylated lncRNA-MIAT DNA probe

MIAT and its antisense RNA were in vitro transcribed and biotin-labeled with the Biotin RNA Labeling Mix (Roche Diagnostics, Indianapolis, IN) and T7/SP6 RNA polymerase (Roche), and purified with an RNeasy Mini Kit (Qiagen, Valencia, CA). The biotinylated lncRNA-MIAT DNA probe was dissolved in binding and washing buffer, and incubated with Dynabeads M-280 Streptavidin (Invitrogen, CA, USA) at room temperature for 10 min to generate probe-coated beads according to the manufacturer’s protocol. Then, LOVO cell lysates were incubated with the probe-coated beads, and the RNA complexes bound to these beads were eluted and extracted for Real-time PCR analysis.

### Pull-down assay with biotinylated miR-132

LOVO cells were transiently transfected with biotinylated miR-132, miR-132-Mut and negative control (Ribobio, Guangzhou, China), harvested and lysed 48 h after transfection. 50 μL of the samples were aliquoted for input. The remaining lysates were incubated with Dynabeads M-280 Streptavidin (Invitrogen, CA, USA) according to the manufacturer’s protocol. In brief, the washed beads were treated in RNase-free solutions and incubated with equal volume of biotinylated miR-132 for 10 min at room temperature in binding and washing buffer on a rotator. Then, the beads with the immobilized miR-132 fragment were incubated with 10 mM EDTA (pH = 8.2) with 95% formamide at 65 °C for 5 min. The bound RNAs were purified using Trizol for the Real-time PCR analysis.

### Luciferase reporter assays

The 3′-UTR of human Derlin-1 or lncRNA-MIAT were amplified from human genomic DNA and individually inserted into the pmiR-RB-REPORT™ (Ribobio, Guangzhou, China) using the XhoI and NotI sites. Similarly, the fragment of Derlin-1 or lncRNA-MIAT 3′-UTR mutant was inserted into the pmiR-RB-REPORT™ control vector at the same sites. For reporter assays, LOVO cells were co-transfected with wild-type (mutant) reporter plasmid and miR-132-Ribo™ mimic (miR-Ribo™ negative control). Luciferase activity was measured 48 h post-transfection as described previously [19].

### Statistical analysis

Statistical analyses were performed using SPSS 16.0 statistical analysis software and were performed using either an analysis of variance (ANOVA) or Student’s *t* test. Data are expressed as mean ± standard deviation. *P* < 0.05 was considered to indicate a statistically significant difference.

## Additional files

**Additional file 1: Figure S1.** Down-regulation of MIAT inhibited SW480 cell proliferation, migration and invasion. SW480 cells were transfected with si-control, si-MIAT-1 or si-MIAT-2 for different time, (A) MIAT expression and cell viability was measured. SW480 cells were transfected with si-control, si-MIAT-1 or si-MIAT-2 for 72 h, (B) cell apoptosis, (C) cell migration and cell invasion was determined. **P < 0.01, compared to si-control.

**Additional file 2: Figure S2..** Down-regulation of MIAT inhibited SW480 cell proliferation, migration and invasion by miR-132/Derlin-1 axis. SW480 cells were transfected with si-MIAT-2, miR-132 inhibitor and Derlin-1 shRNA (shRNA-Derlin-1) for 72 h, (A) cell viability, cell apoptosis, (B) cell migration and cell invasion was determined. **P < 0.01, compared to si-control. ^##^P < 0.01, compared to si-MIAT-2 + NC. & P < 0.01, compared to si-MIAT-2 + miR-132 inhibitor + shRNA.

## Abbreviations

CRC: colorectal cancer
lncRNA-MIAT: long non-coding RNA myocardial infarction associated transcript
miR-132: microRNA-132
ceRNA: competing endogenous RNA
DMEM: Dulbecco’s modified Eagle’s medium
FBS: fetal bovine serum
DC: detergent compatible
DMSO: dimethyl sulphoxide
PI: propidium iodide
ANOVA: analysis of variance
