# Supplementary figures and images for: Long non-coding RNA MIAT promotes growth and metastasis of colorectal cancer cells through regulation of miR-132/Derlin-1 pathway
Source: Cancer Cell Int. 2018 Apr 16;18:59. doi: 10.1186/s12935-017-0477-8 (PMC5902964; doi:10.1186/s12935-017-0477-8)

Figure S1:


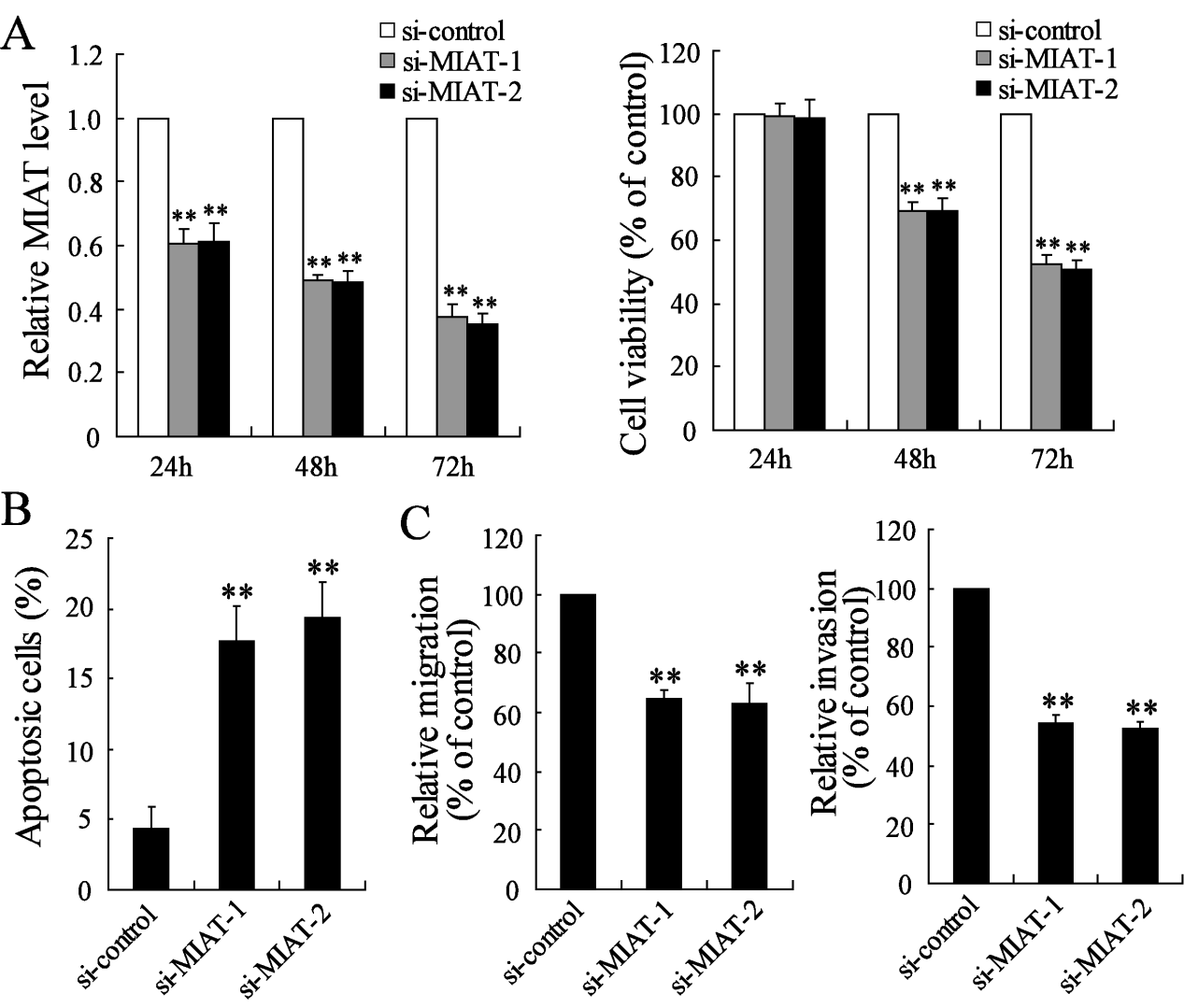

Supplement: Supplementary file 1 — Additional file 1: Figure S1. Down-regulation of MIAT inhibited SW480 cell proliferation, migration and invasion. SW480 cells were transfected with si-control, si-MIAT-1 or si-MIAT-2 for different time, (A) MIAT expression and cell viability was measured. SW480 cells were transfected with si-control, si-MIAT-1 or si-MIAT-2 for 72 h, (B) cell apoptosis, (C) cell migration and cell invasion was determined. **P < 0.01, compared to si-control. [file 12935_2017_477_MOESM1_ESM.docx]

Figure S2


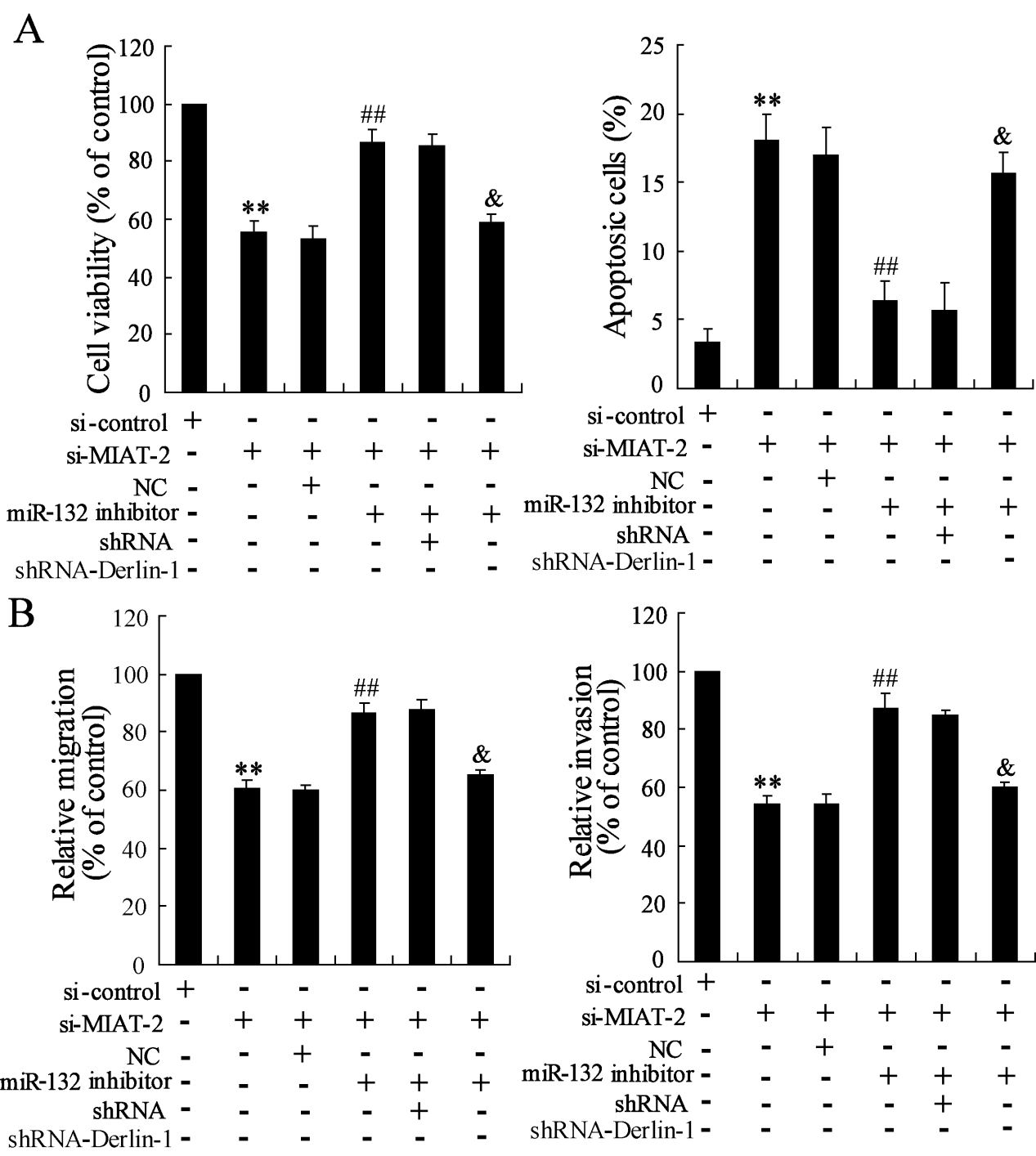

Supplement: Supplementary file 2 — Additional file 2: Figure S2.. Down-regulation of MIAT inhibited SW480 cell proliferation, migration and invasion by miR-132/Derlin-1 axis. SW480 cells were transfected with si-MIAT-2, miR-132 inhibitor and Derlin-1 shRNA (shRNA-Derlin-1) for 72 h, (A) cell viability, cell apoptosis, (B) cell migration and cell invasion was determined. **P < 0.01, compared to si-control. ##P < 0.01, compared to si-MIAT-2 + NC. & P < 0.01, compared to si-MIAT-2 + miR-132 inhibitor + shRNA. [file 12935_2017_477_MOESM2_ESM.docx]
